# The Dufour’s gland and the cuticle in the social wasp *Ropalidia marginata* contain the same hydrocarbons in similar proportions

**DOI:** 10.1093/jis/14.1.9

**Published:** 2014-01-01

**Authors:** A. Mitra, R. Gadagkar

**Affiliations:** 1 Centre for Ecological Sciences, Indian Institute of Science, Bangalore -560012, India; 2 Evolutionary and Organismal Biology Unit, Jawaharlal Nehru Centre for Advanced Scientific Research, Jakkur, Bangalore -560064, India

**Keywords:** cuticular hydrocarbons, fertility signaling, haemolymph

## Abstract

Queens in many social insects are known to maintain their status through chemicals (pheromones) and cuticular hydrocarbons and have been the focus of many investigations that have looked at the chemicals involved in queen signaling. In the primitively eusocial wasp *Ropalidia marginata* Lepeletier (Hymenoptera: Vespidae), the Dufour’s gland has been shown to be involved in queen signaling, and Dufour’s gland hydrocarbons have been found to be correlated with fertility. Hence, this study analyzed the cuticle of *R. marginata* along with the Dufour’s gland in order to compare their hydrocarbon profiles. The results show that the Dufour’s gland and cuticle contained the same set of hydrocarbons in similar proportions (for the majority of compounds). Patterns pertaining to fertility signaling present in cuticular hydrocarbons were also similar to those present in the Dufour’s gland hydrocarbons. Furthermore, the haemolymph contained the same hydrocarbons as found in the Dufour’s gland and cuticle in similar proportions, thereby providing an explanation as to why the hydrocarbon profiles of the Dufour’s gland and cuticle are correlated.

## Introduction

*Ropalidia marginata* Lepeletier (Hymenoptera: Vespidae) is a primitively eusocial paper wasp found in peninsular India. Unlike other typical primitively eusocial wasps, the *R. marginata* queen is a remarkably non-aggressive and non-interactive individual, and thereby cannot use dominance behavior or other behavioral interactions to maintain reproductive monopoly ( Gadagkar 2001 ; Bhadra et al. 2007 ). Therefore, one possibility is that the queen signals her presence to her workers through a pheromone, similar to what is found in highly eusocial species. Recent work emphasizes the Dufour’s gland hydrocarbons as candidates involved in queen signaling or fertility signaling. The Dufour’s gland (DG) has been shown to be at least one source of the queen pheromone, as it has been found that a crude macerate of the queen's DG can act as a proxy for the queen herself, and DG hydrocarbons have been shown to be correlated with the state of ovarian activation of queens ( Bhadra et al. 2010 ; Mitra et al. 2011 ; Mitra and Gadagkar 2011 , 2012a). It has also been shown that the DG composition may act as an honest signal of fertility ( Mitra and Gadagkar 2011 , 2012a). Changes in DG hydrocarbon composition of a potential queen (i.e., future queen) from a worker-like state to a queen-like state, as she undergoes transition to become a new queen of her colony during the queen establishment phase, has also been studied ( Mitra and Gadagkar 2012b ). These evidences highlight the role of the DG in fertility signaling, and it appears that the queen signals her presence to workers by rubbing the tip of her abdomen on the nest surface, thereby presumably applying her DG secretion to the nest ( Bhadra et al. 2007 ). Workers perceive the presence of their queen through her DG compounds and refrain from reproduction, resulting in reproductive monopoly by the queen ( Bhadra et al. 2010 ).

Communication through chemical signals plays an important part in the organization and maintenance of social insect colonies in many species. The major focus of studies investigating chemical communication of fertility status in primitively eusocial species has been on differences in cuticular hydrocarbons (CHCs) between reproductives and non-reproductives ( Bonavita-Cougourdan et al. 1991 ; Peeters et al. 1999 ; Liebig et al. 2000 ; Sledge et al. 2001 ; Dapporto et al. 2005 ). There has been a proliferation in the number of studies looking at CHCs in the last two decades, and CHCs have been im-plicated not only in fertility signaling, but also in nestmate discrimination, dominance signaling, task specific cues, sex pheromones, etc. (reviewed in Howard and Blomquist 2005 ). Thus, CHCs have emerged as an important component of insect chemical communication, having diverse biological roles.

No information was previously available on the CHCs that are present in *R. marginata.* Studies on some bumblebees, such as *Polistes dominula* , *Vespula vulgaris,* hover wasps, and also some solitary wasps, have shown that the DG and cuticle can contain the same compounds ( Oldham et al. 1994 ; Dani et al. 1996 ; Cervo et al. 2002 ; Howard and Baker 2003 ; Bonckaert et al. 2012 ). Hence, to find out the CHCs present in *R. marginata* and to compare them with what is known about the DG compounds, the CHCs and DG hydrocarbons were analyzed. It was found that not only do the cuticle and the DG contain the same compounds in similar proportions (for the majority of compounds), but the patterns related to fertility signaling reported from the DG hydrocarbons are present in the CHCs as well. This is interesting, as CHCs have been implied in fertility signaling in other species (see references cited above), while the DG hydrocarbons are known to perform this role in *R. marginata.* The haemolymph contained the same hydrocarbons as found in the DG and cuticle in similar proportions, thereby providing an explanation as to why the hydrocarbon profiles of the DG and cuticle are correlated.

## Materials and Methods

We report here analysis of CHCs of wasps that were analyzed for Dufour’s gland hydrocarbon composition in earlier studies ( Mitra and Gadagkar 2011 , 2012a, 2012b). Chemical analysis of CHCs was performed simultaneously with analysis of Dufour’s glands, but the CHC data had not been statistically analyzed, interpreted, or published. Post-emergence nests of *R. marginata* were collected from various localities in Bangalore (13° 00’ N, 77° 32’ E), India, and transplanted to the vespiary at the Centre for Ecological Sciences, Indian Institute of Science, Bangalore. Twenty-one nests were used in total. The nests were maintained in closed cages made of wood and fine mesh, and provided with food, water, and building material *ad libitum* . All adults were uniquely color-coded with small spots of Testors® enamel paints on their thorax ( Gadagkar 2001 ). The queen of each colony was identified by observing egg-laying behavior prior to beginning the experiment. For studying solitary foundresses, eight naturally occurring nests founded by solitary foundresses in and around the vespiary were selected.

Analysis of CHCs was done by modification of the protocol followed by Turillazzi et al. (1998) and Sledge et al. (2001) . A small quantity of surgical cotton was wrapped around a toothpick, cleaned by washing in pentane, and dried. The dorsal part of the wasp abdomen was rubbed carefully with the cotton-wrapped toothpick. After rubbing on the abdomen, the cotton was removed from the toothpick, transferred to a glass vial, and CHCs were extracted by washing the cotton bud in 200 mL pentane accompanied by shaking on an orbital shaker for 5 min in chilled condition. After extraction, the cotton was discarded, and the pentane evaporated under a 40 W incandescent table lamp (temperature: approximately 35° C). Then the dry vial was chilled, 10 mL chilled pentane was added to the vial, the vial was then shaken, and 2 mL was drawn out immediately for gas chromatographic analysis. Gas chromatography was done following Mitra et al. (2011) . Blank runs with pentane and cotton-toothpick rinsed pentane (subjected to the same sample preparation procedure as for CHCs) were performed to exclude any contaminants.

For compound identification, sample preparation was performed by pooling together samples from eight wasps, followed by evaporation of the solvent and resuspension of CHCs in 10 mL chilled pentane, as mentioned earlier. This was done to increase the concentration of all compounds, and the same sample was analyzed by gas chromatography as well as gas chromatography coupled with mass spectrometry, following Bhadra et al. (2010) and Mitra et al. (2011) . Identification of compounds was done by interpretation of their mass fragmentation patterns produced by electron impact ionization at 70 eV ( Bhadra et al. 2010 ). Minor peaks that could not be identified were not considered for statistical analysis.

For multivariate analysis, peaks present in at least 70% of all samples were considered. The areas under each peak in an individual were divided by the total area under all peaks in that individual and thereby converted to percent areas ( Bhadra et al. 2010 ; Mitra et al. 2011 ). The transformed data were analyzed by random forest for classification by the package randomForest 4.6-6 in R 2.13.1 ( www.r-project.org ) ( Mitra and Gadagkar 2012b ). The number of trees generated was 100,000, and the number of variables to be used at each decision branch was set to six (recommended to be close to the square root of the number of predictor variables). Random forest is a robust technique that can be used to differentiate individuals using classification trees ( Breiman 2001 ). It does not have any assumptions about the distribution of data, minimum required sample size, etc., and hence should be better at analyzing small sample sizes with a large number of predictor variables than other more conventional techniques ( Cutler et al. 2007 ). The proximities among individuals on the random forest were used to get a measure of Euclidean distance (one minus proximity) between them on the random forest, and the distance matrix was scaled on two dimensions to visualize the relative positions of individuals on the random forest.

As social insects’ hydrocarbon composition data has often been analyzed by discriminant analysis in the literature, and discriminant analysis has also been used earlier to detect fertility related patterns from the DG of *R. marginata* ( Bhadra et al. 2010 ; Mitra et al. 2011 ; Mitra and Gadagkar 2011 , 2012a, 2012b), discriminant analysis was also performed.

*Method of discriminant analysis* : Like analysis by random forest, only those peaks that were identified from the mass spectra and were present in at least 70% of all individuals were used. The areas under each peak in an individual were divided by the total area under all peaks in that individual and thereby converted to percent area. To remove zeroes, 0.001 was added to all zero values, and the resulting data subjected to log ratio transformation ( Aitchison 1986 ; Reyment 1989 ). Levene’s test was performed on each variable to check for homogeneity of variance, and only those peaks that had homogeneous variances were used in discriminant analysis. Further, from the peaks used in discriminant analysis, variables with very low tolerance (< 0.001) were removed from the analysis. The significance of Wilk’s λand percentage of correct classification were used to interpret the results. Since discriminant analysis can lead to overfitting when sample sizes are small, and may result in differences between any groups that are analyzed, permutations were performed to check the robustness of the discriminant analysis ( Dani et al. 2004 , Mitra and Gadagkar 2012b ). Individuals were randomly assigned to any one of the available categories (i.e., queen, worker, solitary foundress, etc., see below), keeping the sample size of each such arbitrary category the same as that in the real data, and the significance of Wilk’s λand percentage of correct classification was calculated. The process was iterated 10,000 times, and the number of times both the significance of Wilk’s λwas < 0.05 and the percentage of correct classification was ≥that in discriminant analysis applied on real data was counted. Thereby the percentage of permutations for which discriminant analysis gave better or equally good discrimination as present in real data was calculated.

The following comparisons were carried out:

*Queen-worker difference* : Six nests were used for looking at the difference between queens and workers. The queen along with six randomly chosen workers had been removed from each nest for analyzing their DG, resulting in six queens and 36 workers in total ( Mitra and Gadagkar 2011 ). Analyses by random forest and discriminant analysis were performed as mentioned above to see if queens and workers can be differentiated and classified correctly based on their CHC composition.

*Solitary foundress* : The CHC profiles of eight solitary foundresses who had founded nests naturally and had laid some eggs were studied. The CHC profiles of solitary foundresses were compared with 12 queens and 36 workers (the same six queens and 36 workers as above, and another six additional queens from other nests) from normal post-emergence nests, as had been done for the Dufour’s gland data ( Mitra and Gadagkar 2012a ), by random forest and discriminant analysis, as mentioned above. Distances between individuals on the random forest and in the discriminant functions space were analyzed by Mann-Whitney *U* tests.

*Transition of potential queen to queen* : When an *R. marginata* queen is removed from her colony, one of the workers starts showing high levels of aggression, and if the queen is not returned, she gradually loses her aggression, activates her ovaries, and becomes the new queen of the colony within a few days of queen removal. This individual is called the potential queen or PQ (Chandrashekara and Gadagkar 1992; Premnath et al. 1996 ; Deshpande et al. 2006 ). The change in Dufour’s gland composition of the PQ from a worker-like state to a queen-like state, as a function of time since queen removal, has been shown previously ( Mitra and Gadagkar 2012b ). In our study the CHCs of these same PQs were analyzed in comparison with queens and workers to see if the CHC profile of PQs shows similar dynamics as seen for the DG hydrocarbons. The PQ along with six randomly chosen workers were collected from different colonies either immediately after queen removal (day 0), three days after queen removal (day 3), or five days after queen removal (day 5) (three PQs per category, total sample size = nine PQs, nine queens and 54 workers from nine different colonies). The experimental protocol for collecting PQs at different stages of the queen establishment phase (when PQ undergoes transition to become the new queen of her colony) has been mentioned in details in Mitra and Gadagkar (2012b) . Random forest and discriminant analysis were applied as mentioned earlier to look at change in CHC profiles of PQs in comparison with CHC profiles of queens and workers. Distances between PQs and queens on the random forest and on the discriminant functions space were analyzed by Mann-Whitney *U* tests.

The area under each peak was converted to a percentage by dividing with the total area under all peaks in an individual, and chemical distances were calculated by computing squared Euclidean distances between all possible pairs of individuals (all PQs, queens, and workers) using standardized percentages of areas under the peaks (standardization done by taking Z scores) ( Bhadra et al. 2010 ; Mitra and Gadagkar 2012b ). Chemical distance gives the distance between individuals in n dimensional space and thus can be used to estimate how close or far apart individuals are in n dimensions. Chemical distances between PQs and queens were compared with distances between PQs and workers for each day (day 0, day 3, and day 5) by Mann-Whitney *U* tests.

*Correlation between CHCs and DG hydrocarbons* : Because the compounds identified from the cuticle were the same as those identified from the DG, and the CHC profile of each wasp generally appeared to be similar to the DG profile by visual inspection, a reduced major axis regression was performed, following Strohm et al. (2010) , using mean percent area under each peak for DG and CHCs. This was done using data from the six queens and 36 workers mentioned above to see if there was a correspondence between the overall pattern of peaks found in the DG with those found from the cuticle. Spearman’s rank correlation analysis was performed for each peak, one at a time, to see if the relative abundance of each peak in an individual was the same for both DG and cuticle. To find out if there was any difference between CHC and DG hydrocarbon profiles, the data was subjected to analysis by random forest and discriminant analysis as mentioned earlier.

Because it has been reported that insect hydrocarbons are synthesized in oenocytes of fat bodies, from where they are transported to various parts of the body through carrier proteins of the haemolymph ( Schal et al. 1998 ), gas chromatography analysis of the haemolymph was performed to investigate whether the hydrocarbons present in the cuticle and the DG were present in the haemolymph as well. An additional 15 wasps collected from different colonies were utilized for this analysis. Each wasp was anesthetized by chilling on ice, and after extraction of CHCs (done as mentioned above), was centrifuged at 4° C in an upright position at 1000 rpm for 10 min, attempting to maximize the amount of haemolymph present in the abdomen. Then the abdominal segments were gently pulled apart with a forceps, a micropipette tip was inserted through the gap between two segments, and the haemolymph drawn out. The haemolymph was immediately added to 100 µL chilled pentane, which was subjected to centrifugation at 4° C at 4000 rpm for 10 min so as to remove all debris insoluble in pentane. The supernatant was transferred to a glass insert, and the solvent was evaporated under a 40 W table lamp, as was done for CHCs. After evaporation of solvent, the dry insert was chilled and another 10 µL of chilled pentane was added to the insert, of which 2 µL was used for gas chromatography analysis. Gas chromatography conditions were the same as for analysis of CHCs and DG hydrocarbons. Finally, the DG of each wasp was dissected and analyzed by gas chromatography following Mitra et al. (2011) .

The retention times and patterns of peaks obtained from haemolymph was compared with those obtained from the cuticle and DG, and peaks of the haemolymph whose retention times were identical with those of known peaks from the cuticle and DG were selected for statistical analysis. Because the hydrocarbon profile of the haemolymph of each wasp generally appeared to be similar to the DG and CHC profile by visual inspection, reduced major axis regressions were performed using mean percent area under each peak for the DG, CHCs, and haemolymph to see if the overall pattern of hydrocarbon peaks from the cuticle, DG, and haemolymph were similar ( Strohm et al. 2010 ).

Statistical analyses were done using the software packages StatistiXL 1.7 ( www.statistixl.com ) and R 2.13.1.

## Results

**Figure 1.**
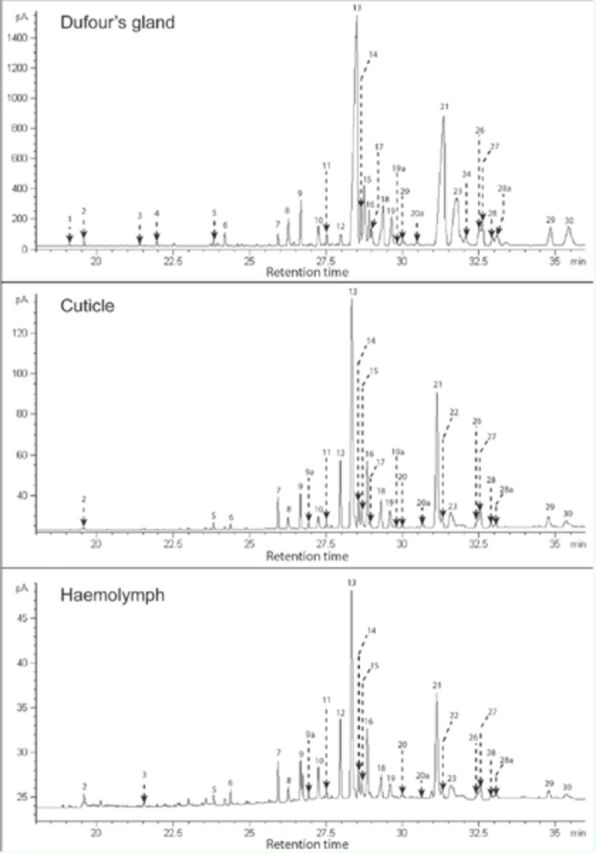
Flame ionization detection gas chromatograms of Dufour’s gland, cuticle, and haemolymph of an individual *Ropalidia marginata* wasp. Peak numbers correspond with compounds identified by GC-MS ( Table 1 ). High quality figures are available online.

The CHC profile of each *R. marginata* wasp was similar to the DG profile. All of the 34 peaks that had been identified earlier from the DG were identified from the cuticle as well. The retention time and the mass fragmentation pattern of each peak from the cuticle were identical to its counterpart from the DG, and the relative proportions of the peaks were also similar ( Figure 1 , Table 1 ). There were no peaks found from the cuticle that were in addition to what had already been reported from the DG ( Bhadra et al. 2010 ; Mitra et al. 2011 ). As reported for the DG compounds, the cuticular compounds were linear, monomethyl, and dimethyl branched alkanes (21 to 33 carbon atoms in the main chains), with some peaks comprising a mixture of two or more positional isomers of a branched alkane ( Table 1 ).

**Table 1.**
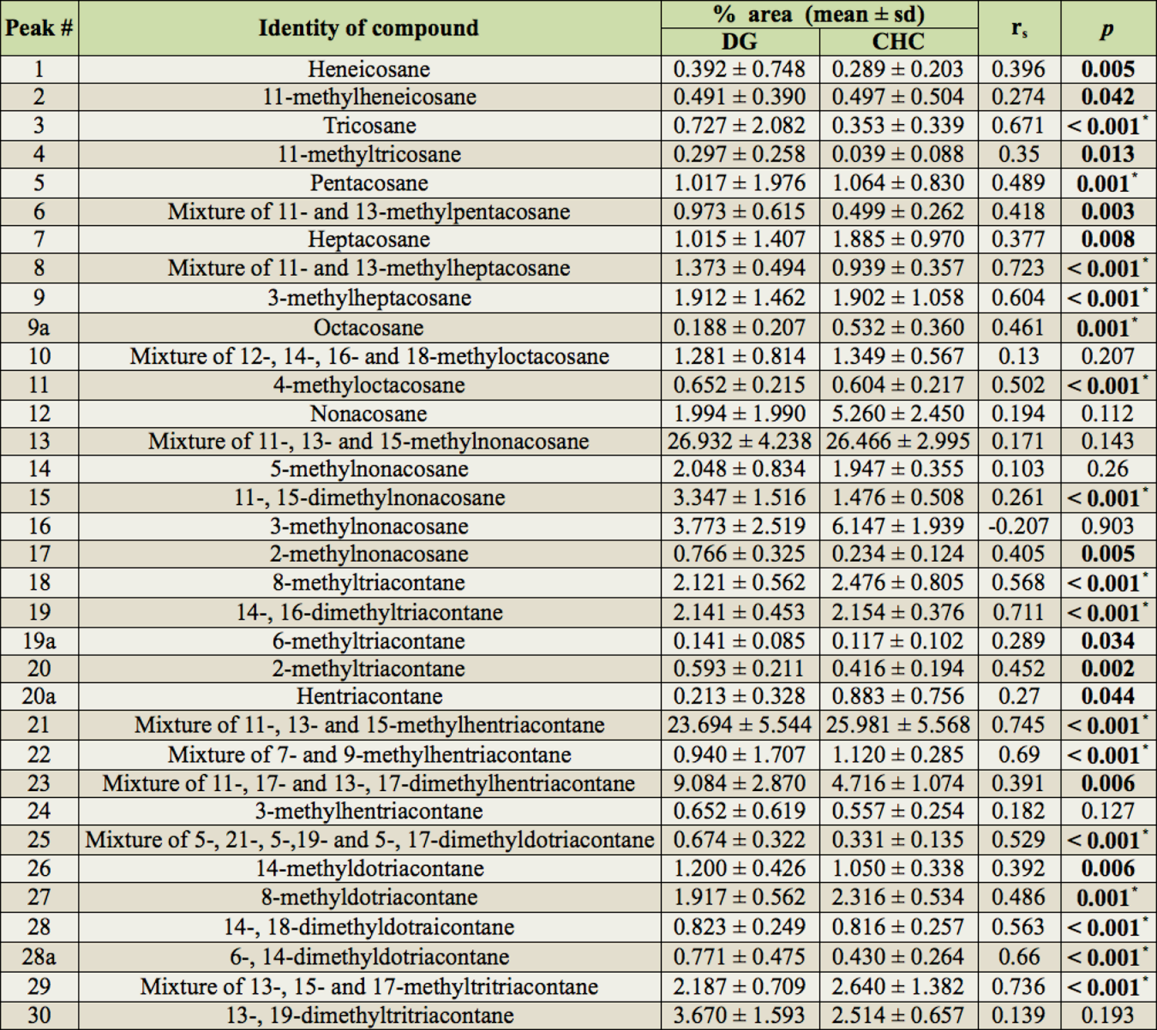
Peak numbers, identities, percent area (mean ± standard deviation) and correlations between compounds found in gas chromatographic analysis of Dufour’s glands (DG) and cuticular hydrocarbons (CHC) of *Ropalidia marginata.* Compounds identified by gas chromatography-mass spectrometry. Significant *p* -values for Spearman’s rank correlation (rs) between Dufour’s glands and cuticle for each compound are highlighted in bold. Asterisk signifies *p* -values that remain significant after Bonferroni correction ( *p* critical = 0.0015).

**Figure 2.**
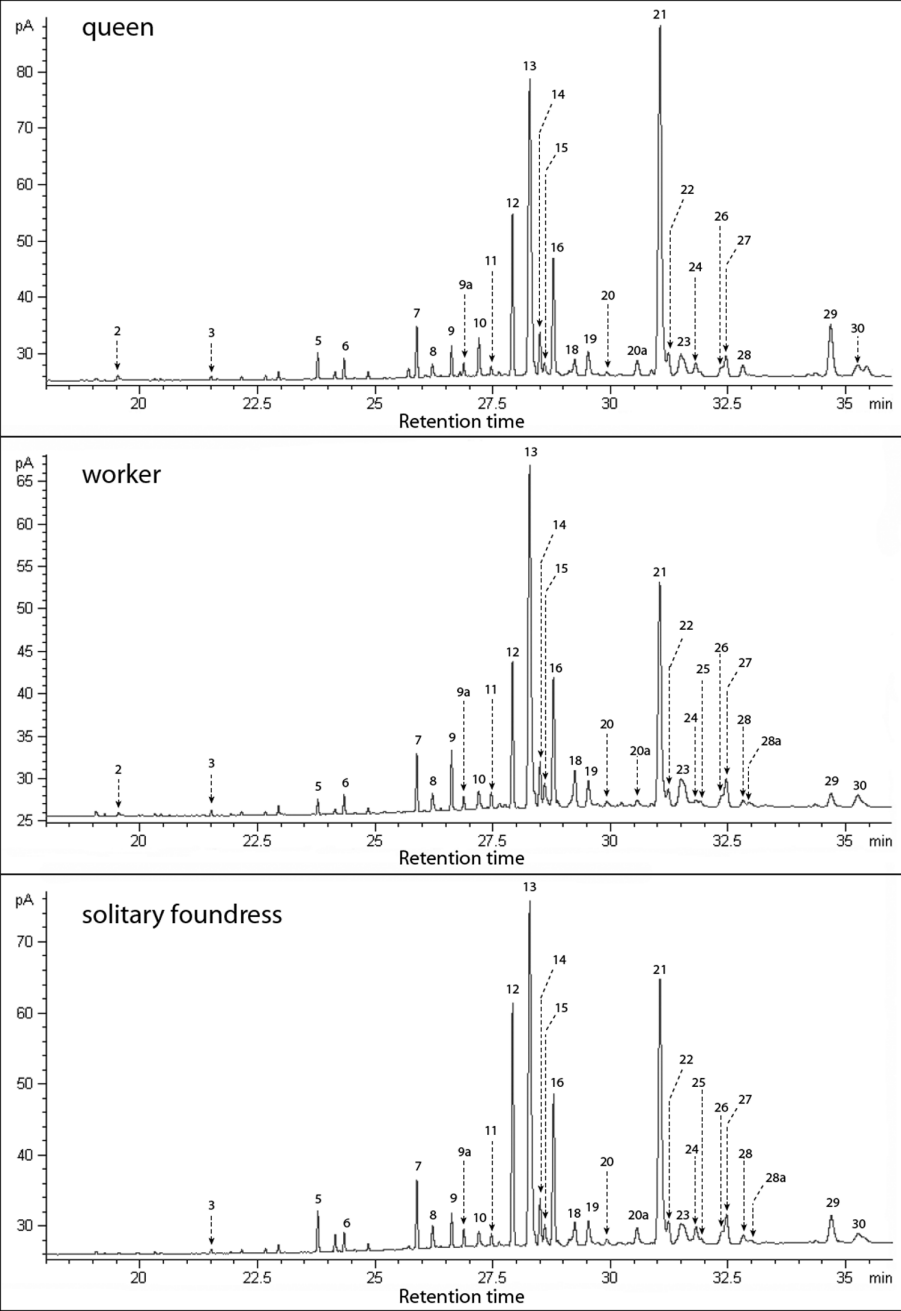
Flame ionization detection gas chromatograms of cuticular hydrocarbons of a queen, a worker, and a solitary foundress of *Ropalidia marginata* . High quality figures are available online.

**Figure 3.**
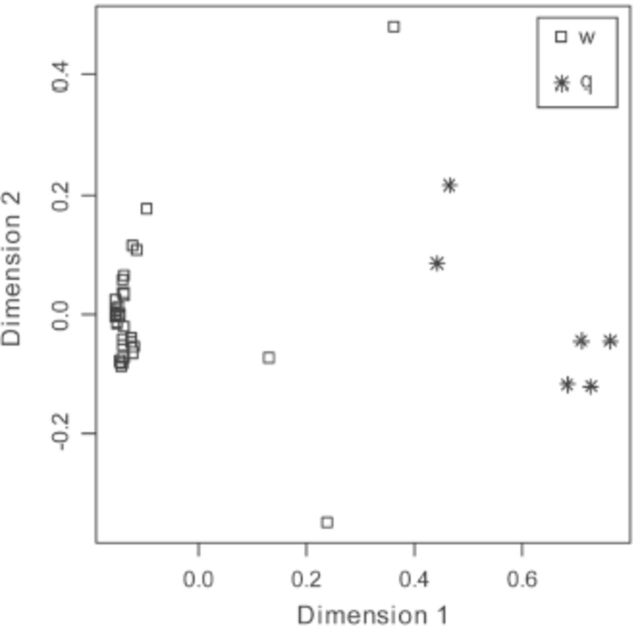
Proximities among queens (q) and workers (w) of *Ropalidia marginata* (N = 6 and 35 respectively) on a random forest (100,000 trees, number of randomly selected variables used at each branch = 6) that differentiates queens and workers based on relative abundances of 32 hydrocarbon peaks from the cuticle. The proximity distances have been scaled on two dimensions. High quality figures are available online.

*Queen-worker difference* : The overall CHC profiles were similar for queens and workers ( Figure 2 ), and no compound or set of compounds present exclusively in queens or in workers were found. One worker had most CHCs below detection limit of the gas chromatography, and hence was kept out of analysis. Queens and workers formed separate clusters on the random forest (N = 6 queens, 35 workers) ( Figure 3 ). Peaks 4 and 19a were kept out of this analysis (present in < 70% samples).

Discriminant analysis gave similar results. Wilk’s λ= 0.011, *p* < 0.001, classification analysis: 100% correct classification. Randomly assigning individuals to categories and running discriminant analysis showed that discrimination as good as or better than that on real data was obtained only in 7.54% of permutations (out of 10,000 permutations), suggesting that the results of discri-discriminant analysis performed on real data are likely to be robust.

**Figure 4.**
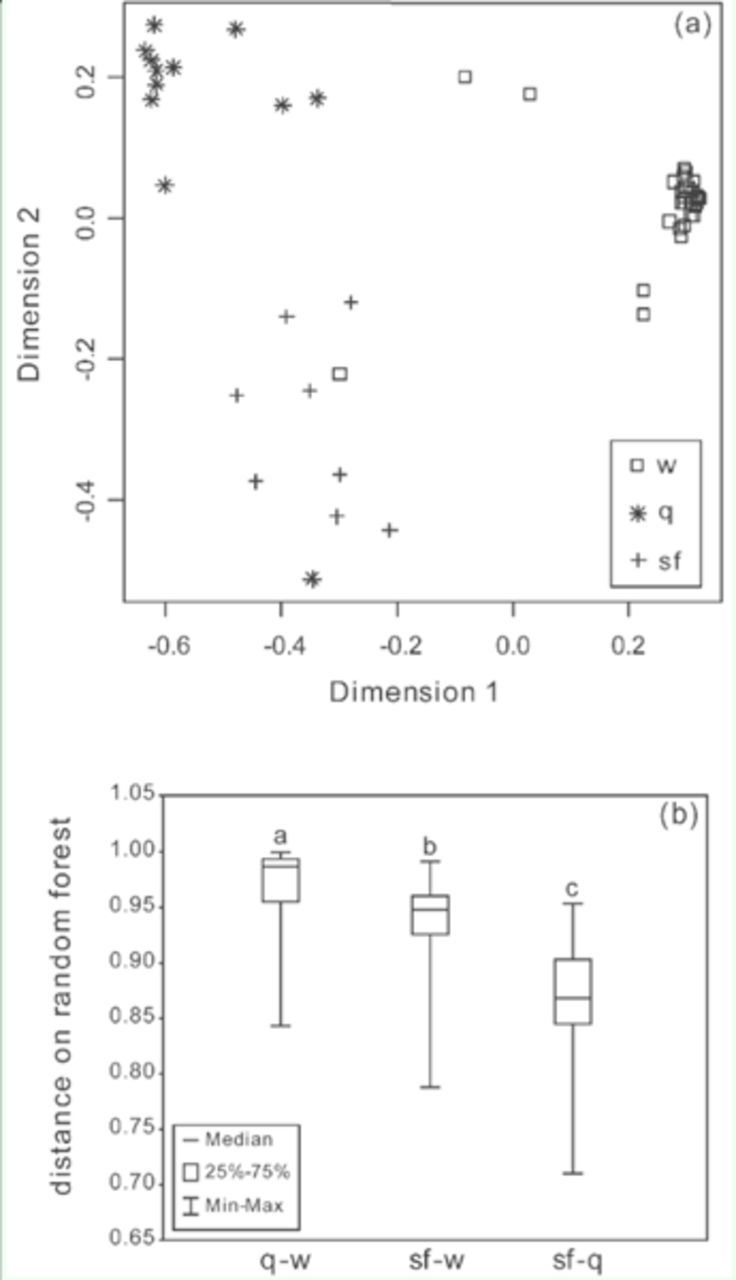
(a) Proximities among queens (q), solitary foundresses (sf), and workers (w) of *Ropalidia marginata* (N = 12, 8, and 35 respectively) on a random forest (100,000 trees, number of randomly selected variables used at each branch = 6) that differentiates individuals based on relative abundances of 31 hydrocarbon peaks from the cuticle. The proximity distances have been scaled on two dimensions. (b) Distance on random forest between queens and workers (q-w), solitary foundresses and workers (sf-w), and solitary foundresses and queens (sf-q). Different letters denote a significant difference between distributions (Mann-Whitney *U* test, *p* < 0.05, N = 420, 280, and 96 respectively). High quality figures are available online.

*Solitary foundress:* The CHC profiles of solitary foundresses were qualitatively similar to those of queens and workers of post-emergence nests ( Figure 2 ). Solitary foundresses, queens, and workers appeared to form three clusters on the random forest (N = 8 solitary foundresses, 12 queens, and 35 workers) ( Figure 4a ). One queen and one worker were found to lie in the solitary foundress cluster. Peak 4, 19a, and 28a were kept out of this analysis (present in < 70% samples). The distance between individuals on the random forest showed that distances between queens and workers were significantly greater than distances between both solitary foundresses and queens (Mann-Whitney *U* test, *U* = 39153, *p* < 0.001, N = 420 and 96) and solitary foundresses and workers ( *U* = 91422, *p* = 0, N = 420 and 280). The distances between solitary foundresses and queens were significantly lower than distances between solitary foundresses and workers ( *U* = 25170, *p* < 0.001, N = 96 and 280). This shows that though solitary foundresses are intermediate between queens and workers, forming a separate cluster, they are closer to queens than to workers ( Figure 4b ).

The results of discriminant analysis were similar to those of random forest. Discriminant function 1: Wilk’s λ= 0.005, *p* < 0.001; discriminant function 2: Wilk’s λ= 0.142, *p* < 0.001; classification analysis: 100% correct classification. Randomly assigning individuals to categories and running discriminant analysis showed that discrimination as good as or better than that on real data was obtained not even once out of 10,000 permutations, suggesting that the results of discriminant analysis performed on real data are likely to be robust.

**Figure 5.**
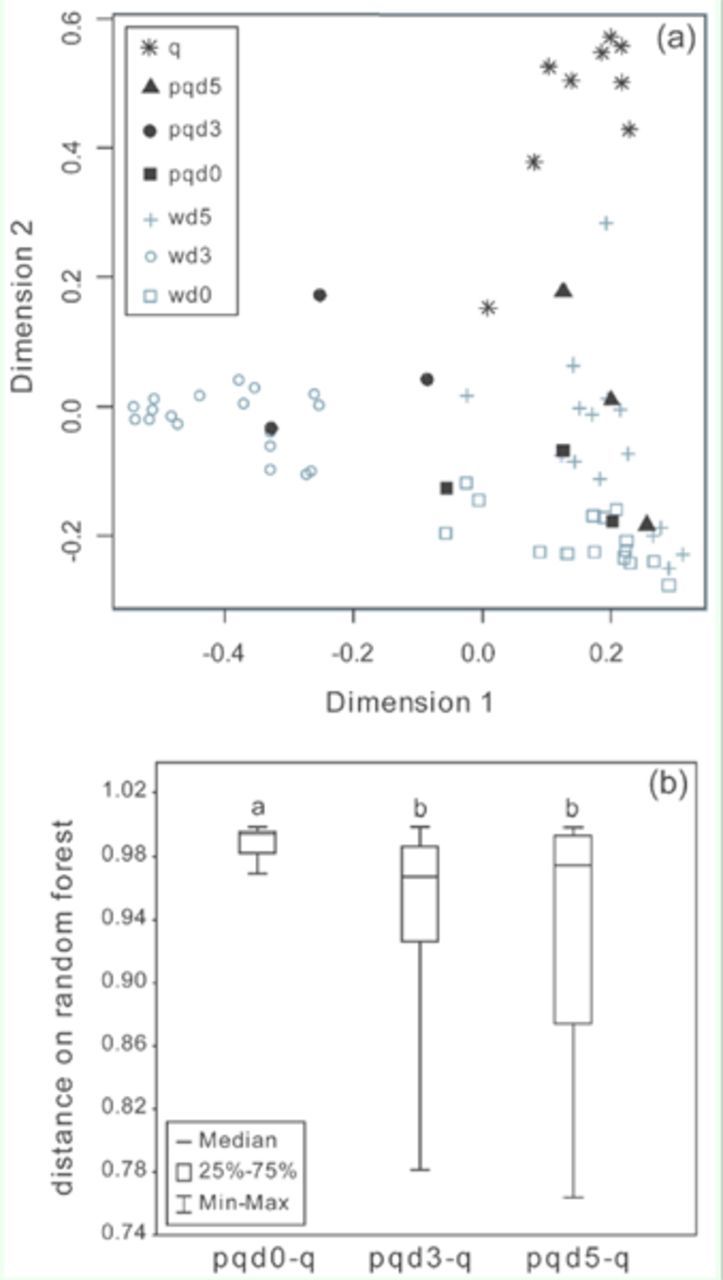
(a) Proximities among *Ropalidia marginata* individuals on a random forest (100,000 trees, number of randomly selected variables used at each branch = 6) that differentiates individuals based on relative abundances of 31 hydrocarbon peaks from the cuticle. The proximity distances have been scaled on two dimensions. Abbreviations: q: queen, pqd5: day 5 PQ, pqd3: day 3 PQ, pqd0: day 0 PQ, wd5: day 5 worker (N = 9 q, 3 pqd5, 3 pqd3, 3 pqd0, 16 wd5, 18 wd3, and 17 wd0). (b) Distance on random forest between day 0 PQs and queens (pqd0-q), day 3 PQs and queens (pqd3-q), and day 5PQs and queens (pqd5-q). Different letters denote a significant difference between distributions (Mann-Whitney *U* test, *p* < 0.05, N = 27 for each distribution). High quality figures are available online.

*Transition of potential queen to queen* : One day 0 worker and two day 5 workers were found to have most compounds below the detection limit of gas chromatography and hence were kept out of analyses. Analysis by random forest showed that PQs’ compounds were similar to workers in the beginning (queen-right condition, i.e. day 0), occupying a position in the middle of all workers, and after queen removal appeared to gradually come closer to queens with passage of time (N = 9 queens, 3 PQs each for day 0, day 3, and day 5; 17 day 0 workers, 18 day 3 workers, and 16 day 5 workers) ( Figure 5a ). Some workers also appeared to be moving closer to queens, and this could be because some workers were observed to spend a large proportion of time away from the nest after queen removal, and also started developing their ovaries ( Mitra and Gadagkar 2012b ). Peaks 4, 17, and 19a were kept out of this analysis (present in < 70% samples). The distance between individuals on the random forest showed that distances between day 0 PQs and queens were higher than both distances between day 3 PQ's and queens ( *U* = 599, *p* < 0.001, N = 27 for both distributions) and day 5 PQs and queens ( *U* = 553, *p* < 0.001, N = 27 for both distributions). Distances between day 3 PQs and queens were not different from distances between day 5 PQs and queens ( *U* = 386, *p* = 0.719, N = 27 for both distributions). This suggests that amongst all PQs, day 0 PQs were furthest away from queens, while day 3 and day 5 PQs were closer to queens ( Figure 5b ).

Discriminant analysis gave similar results. Out of six discriminant functions, functions 1 and 2 were significant (function 1: Wilk’s λ=0.001, *p* < 0.001; function 2: Wilk’s λ= 0.012, *p* < 0.001; N = nine queens, three PQs each for day 0, day 3, and day 5, 17 day 0 workers, 18 day 3 workers, and 16 day 5 workers). Classification analysis gave 98.6% overall correct classification. Randomly assigning individuals to categories and running discriminant analysis showed that discrimination as good as or better than that on real data was obtained not even once out of 10,000 permutations, suggesting that the results of discriminant analysis performed on real data are likely to be robust.

Analysis of chemical distance showed that on day 0, PQ-worker distances were lower than PQ-queen distances ( *U* = 932, *p* = 0.005), and on day 3 PQ-worker distances were lower than PQ-queen distances ( *U* = 1159, *p* < 0.001). The difference between PQ-worker and PQ-queen distances was not significant on day 5 ( *U* = 740, *p* = 0.315), implying that for days 0 and 3, PQs were closer to workers than to queens, but were equidistant from workers and queens on day 5, showing that PQs are coming closer to queens with time since queen removal.

*Correlation between CHCs and DG hydrocarbons* : Reduced major axis regression was done for the mean values for percent area under the peaks for DG versus the corresponding values for cuticle to look at the overall similarity between the two profiles. It was expected that if there is a one to one correspondence between DG and cuticle for relative abundance of all peaks, the slope of the regression should be equal to one and the intercept should be equal to zero ( Strohm et al. 2010 ). The results were: slope = 1.027, 95% confidence interval of slope = 0.961 to 1.093; intercept = -0.081, 95% confidence interval of intercept = -2.961 to 1.093; R ^2^ = 0.96; N = 41. This shows that the slope was not significantly different from one and the intercept was not significantly different from zero, as both one and zero lie within the 95% confidence limit of each statistic, respectively. The overall correlation between the DG and CHC profiles was also quite high (R ^2^ = 0.96). The test for correlation between DG and cuticle for each peak, one at a time, showed that out of 34 peaks, 27 peaks were significantly positively correlated (Spearman’s rank correlation *,p* < 0.05, N = 41 for each peak). After Bonferroni correction ( *pcritical* = 0.0015), 16 out of these 27 correlations remained significant ( Table 1 ).

**Figure 6.**
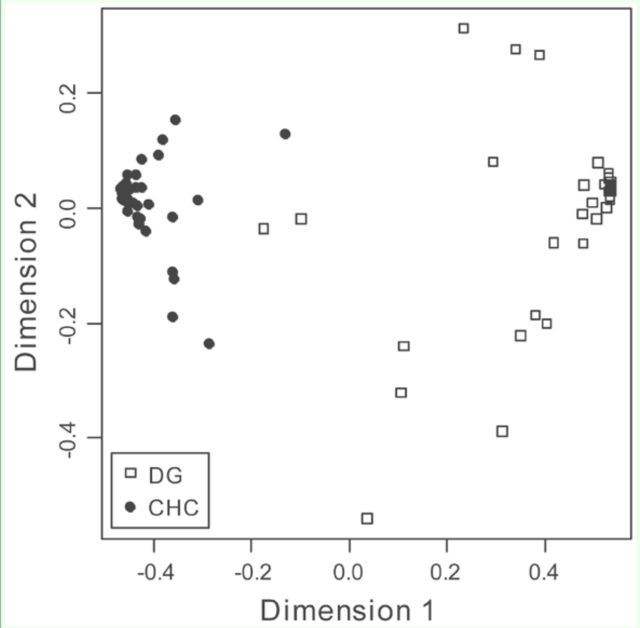
Proximities among points on a random forest (100,000 trees, number of randomly selected variables used at each branch = 6) that differentiates Dufour’s gland (DG) and cuticular hydrocarbon (CHC) profiles of *Ropalidia marginata* individuals based on relative abundances of 33 hydrocarbon peaks (N = 41and 41). The proximity distances have been scaled on two dimensions. High quality figures are available online.

The CHC and DG hydrocarbon profiles could be differentiated using random forest (N = 41 for both DG and CHC) ( Figure 6 ). Peak 4 was removed from this analysis (present in < 70% samples). From the results of the random forest, it appears that DG profiles were more variable than CHC profiles. DG and CHC profiles could also be differentiated using discriminant analysis.

The retention times and relative abundances of peaks obtained from the haemolymph were similar to those found in the DG and CHCs ( Figure 1 ). Reduced major axis regressions showed that the overall pattern of peaks in haemolymph, DG, and cuticle were similar (N = 15 individuals for each category). Regression between DG and haemolymph: slope = 0.98, 95% confidence interval of slope = 1.084 to 0.875; intercept = 0.059, 95% confidence interval of intercept = 6.548 to -6.429; R ^2^ = 0.78. Regression between CHCs and haemolymph: slope = 1.004, 95% confidence interval of slope = 1.113 to 0.895; intercept = -0.011, 95% confidence interval of intercept = 6.713 to -6.734; R ^2^ = 0.76. Regres-Regression between DG and CHCs: slope = 0.976, 95% confidence interval of slope = 1.077 to 0.875; intercept = 0.07, 95% confidence interval of intercept = 6.115 to -5.975; R ^2^ = 0.81. Thus it can be seen that the relative abundances of hydrocarbons present in the haemolymph were similar and correlated with that of the cuticle and DG.

## Discussion

There is growing evidence for CHCs as correlates and signals of fertility in various social insects, both primitive as well as highly eusocial ( Bonavita-Cougourdan et al. 1991 ; Peeters et al. 1999 ; Liebig et al. 2000 ; Sledge et al. 2001 ; D’Ettorre et al. 2004 ; Dapporto et al. 2005 ; Monnin 2006 ). Our previous work highlighted the role of the DG hydrocarbons in fertility signaling in *R. marginata* ( Bhadra et al. 2010 ; Mitra et al. 2011 ; Mitra and Gadagkar 2011 ; Mitra and Gadagkar 2012a , b) *.* The present study showed that not only did the DG and cuticle contain the same hydrocarbons, but patterns related to fertility signaling that have been previously found in the DG hydrocarbons could be found in CHCs as well, and there was also a positive correlation between the CHC profile and DG profile with respect to relative proportions of compounds for the majority of compounds. The results are rem-iniscent of what has been found with respect to DG and CHCs in *Vespula vulgaris* ( Bonckaert et al. 2012 ).

Queens and workers could be differentiated using their CHC profiles. Solitary foundresses were intermediate between queens and workers in their CHC profiles. Though these solitary foundresses had started laying eggs, they have been shown to have ovaries intermediate between those of queens and workers ( Mitra and Gadagkar 2012a ). Thus, the CHC profile of *R. marginata* individuals can be linked to their state of ovarian development. The CHCs of PQs showed similar dynamics, as seen in the DG hydrocarbons ( Mitra and Gadagkar 2012b ). The CHC profile of PQs were similar to workers immediately after queen removal, but gradually appeared to come closer to that of queens with passage of time.

Earlier work on *R. marginata* showed that the DG macerate of the queen can mimic the queen herself in a bioassay, showing that the DG is at least one source of the queen pheromone in this species ( Bhadra et al. 2010 ; Mitra et al. 2011 ). Earlier work also showed that since the *R. marginata* queen is remarkably non-interactive, it should not be possible to convey the queen signal from queen to workers through direct or indirect interactions with the queen ( Bhadra et al. 2007 ). Hence, CHCs are apparently unlikely to be candidates involved in conveying the queen signal, as it should not be possible to perceive any signals carried by CHCs without physically interacting with the queen. However, the patterns of fertility signaling found in the DG hydrocarbons are being found in the CHCs as well, and the hydrocarbon profile of the DG and cuticle are correlated with each other and also correlated with the haemolymph hydrocarbon profile, suggesting a common source of hydrocarbons for the DG and cuticle. Interestingly, despite the overall similarity between DG and CHC profiles, they could also be differentiated using multivariate supervised classification techniques. This could be due to minor differences between the DG hydrocarbons and CHCs in percent areas under a few peaks ( Table 1 ). It is not clear at this stage whether this difference can be interpreted as difference in information conveyed to the wasps through DG and cuticle.

A positive correlation was found between DG hydrocarbon and CHC profiles, both in overall composition as well as in percent area under each peak (for the majority of peaks). The same pattern of peaks was found from the haemolymph as well, and the pattern was positively correlated with the hydrocarbon peak patterns found in the DG and CHCs. This finding is consistent with the framework of hydrocarbons being synthesized in oenocytes of fat bodies and then being transported through haemolymph carrier proteins (lipophorin) to other body parts ( Schal et al. 1998 ; Schal et al. 2001 ; Fan et al. 2002 ; Bagnères and Blomquist 2010 ). Thereby both the cuticle and the DG may uptake or sequester hydrocarbons being carried in the haemolymph. Recently it has been suggested that the postpharyngeal gland in a solitary wasp may sequester hydrocarbons from the haemolymph ( Strohm et al. 2010 ). The results of our study suggest that the DG also may sequester hydrocarbons from the haemolymph and stimulate further thought on hydrocarbon sequestration mechanisms in exocrine glands of insects. Thus, for any change in the hydrocarbon profile related to fertility status and fertility signaling between individuals, the change is likely to occur at the hydrocarbon production site itself, and physiological factors that affect fertility signaling by hydrocarbons are thereby likely to act on the site of hydrocarbon synthesis itself (oenocytes), with the cuticle and DG acting as portals for conveying the information present within the haemolymph. Since the hydrocarbon profiles of the DG and cuticle were not significantly correlated for a few peaks ( Table 1 ), this difference could arise from differences in sequestration of hydrocarbons from haemolymph by the DG and cuticle.

It is concluded that the hydrocarbons of an *R. marginata* female can be synthesized inside the body (in the oenocytes) and are carried by the haemolymph (probably bound to some carrier protein like lipophorin) to the DG and cuticle. This explains why the patterns related to fertility signaling seen in the DG hydrocarbons are manifested in the CHCs as well.

## Abbreviations

CHC: cuticular hydrocarbon
DG: Dufour’s gland
PQ: potential queen
